# African American women's perceptions of the meaning of support groups for improving adherence to hypertension treatment: a conceptual model

**DOI:** 10.1002/nop2.266

**Published:** 2019-04-01

**Authors:** Marie N. Fongwa, Felicitas A. dela Cruz, Ron D. Hays

**Affiliations:** ^1^ School of Nursing Azusa Pacific University Azusa California; ^2^ Department of Medicine UCLA Los Angeles California

**Keywords:** African American women with high blood pressure, conceptual model of support groups, facilitators and barriers to joining support groups, forming support groups, meaning of support groups, resources exchanged in social relationships, types of social support

## Abstract

**Aim:**

To investigate the meaning of support groups and the features of these groups that African American (AA) women view as improving adherence to high blood pressure (HBP) treatment. The study generated a conceptual model to illuminate features of these groups that influence adherence of AA women to HBP treatment.

**Design:**

Qualitative research.

**Methods:**

Used focus groups and open‐ended questions to obtain the views of 26 eligible AA women, recruited from South Los Angeles. Line‐by‐line review and coding of interview transcripts were done. The feedback was used to specify a conceptual model depicting the meaning of support groups. The Consolidated Criteria for the Reporting of Qualitative Research guidelines were used.

**Results:**

The conceptual model depicts the meaning of support groups as information giving/knowledge sharing, emotional or psychological support, instrumental support and coaching, and facilitators and barriers to joining support groups and factors for consideration in forming these groups.

## 1 INTRODUCTION

Hypertension or high blood pressure (HBP), a major contributor to cardiovascular disease and stroke, affects about 78 million persons in the United States (Go et al., 2014). The HBP prevalence rate of Black/African Americans (AAs) is one of the highest of any subgroup in the world. National Health and Nutrition Examination Survey (NHANES) data from 2007–2012 indicate that AA women, age ≥ 20 years, have a higher prevalence than AA men (43% vs. 40%) and White men (30%) and women (28%). Adherence is challenging for those with HBP and is especially poor for AAs (Bosworth et al., 2006; Lewis, Ogedegbe, & Ogedegbe, 2012). Ritchey et al. (2016) reported non‐adherence to HBP medications in AAs to be 36% compared with 24% in Whites and 26% in Asian Pacific Islanders. Antihypertensive non‐adherence, defined as a proportion of days a beneficiary was covered with antihypertensives of <80%, was assessed using prescription drug claims data among Medicare Advantage or Medicare fee‐for‐service beneficiaries aged ≥ 65 years with Medicare Part D coverage during 2014 (Ritchey et al., 2016). The rate of HBP control for AA women is only about 53% (Go et al., 2014). Based on the U.S. Department of Health and Human Services' (U.S.D.H.H.S., 2016) report, non‐adherence to needed medications for HBP is the main cause of poor management of the disease.

## 2 BACKGROUND

African Americans face socio‐economic issues that contribute to the high prevalence and lack of control of HBP: less educational attainment, lower household income and lack of health insurance coverage (Office of Minority Health, 2014). One of the strongest determinants of morbidity and mortality is socio‐economic status (Dubay & Lebrun, 2012; Mansyur, Pavlik, Hyman, Taylor, & Goodrick, 2013; Pampel, Krueger, & Denney, 2010). For AAs with hypertension, lower socio‐economic status is associated with more unhealthy behaviours and poorer health outcomes (Dubay & Lebrun, 2012; Mansyur et al., 2013). There is a lack of approaches to increase adherence to treatment for HBP in inner‐city and underserved communities (Kotchen, Shakor‐Abdullah, Walker, Peters, & Kotchen, 1996; Morisky, Bowler, & Finlay, 1982; Yi et al., 2015). However, inner‐city AA women with HBP identified support groups as desirable for enhancing their adherence to treatment (Fongwa et al., 2008). Support groups are a gathering of people with similar health problems who meet to share experiences, ask questions, give practical advice or provide encouragement and self‐help (WebMD, 2018). Support groups serve as a resource for social support, embodying social interactions, relationships and exchanges. However, the specific elements of support groups that AA women find beneficial are not well understood. Therefore, this study investigated the meaning of support groups and their features that AA women view as improving adherence to HBP treatment. Specifically, the study used focus group interviews to learn from the participants the (a) meaning of HBP support groups, (b) factors that facilitate or hinder their joining support groups and (c) factors to consider in forming these groups.

### 2.1 Theoretical perspective

We used social constructivism as our theoretical perspective. Social constructivism posits that people derive knowledge and meanings through interactions with others (Mckinley, 2015). In addition, the constructed knowledge and meanings correspond to individual's experiences with others and the environment they interact with (Ponterotto, 2005). Social constructivism suggests that multiple realities exist in the minds of individuals that emerge, surface and are shared through social interactions and social processes (Creswell, 2013). Focus groups uncover feelings, perspectives and issues and can provide richer information than personal interviews or surveys because group dynamics lead to more developed responses than any one person can provide.

## 3 METHODS

### 3.1 Research design, participants and settings

The planning and execution of the study, including data analysis, interpretation and reporting of findings, adhered to the Consolidated Criteria for the Reporting of Qualitative Research (COREQ) guidelines (Tong, Sainsburry, & Craig, 2007). We conducted focus groups to obtain data from 26 inner‐city AA women with hypertension, recruited from a federally funded health clinic in South Los Angeles. Eligibility for the study included self‐identified AA woman 21 years or older and prescribed antihypertensive medication.

The study was advertised at the clinic and surrounding neighbourhoods using the University Institutional Review Board‐approved flyer. Azusa Pacific University received from the authors and reviewed the study protocol (approval # IRB 07–12). The flyer stated the eligibility criteria and asked interested study persons to provide contact information. We included some individuals without previous experience to help identify what focus group naïve women desired. The flyer also included the contact information, name and phone number of the principal investigator (PI), and interested participants were asked to call the number. Those who called either spoke directly with the PI or left a telephone voice message. Those who left messages were called back. The PI and research assistant (RA) used a recruitment log to document the names and contact information of interested individuals. The recruitment log was kept in a locked cabinet at the PI's research office. Each participant was contacted via U.S. mail with the date, time and place for a focus group meeting. A reminder telephone call was made a day prior to the meeting date. All four focus group sessions were held at a convenient place in a nearby recreation centre and close to the federally funded health centre.

### 3.2 Data collection

The sample size for the four focus groups in this study ranged from 6–7 participants per group. Focus groups were held late mornings or early afternoons on weekdays and lasted for 1½ to 2 hr. Refreshments were served, and each participant received $30 cash in appreciation for her participation.

#### 3.2.1 Measures

At the start of the focus groups, each participant was asked to complete a demographic and clinical data sheet and the Facilitators of and Barriers to Adherence to Hypertension Treatment Scale (FATS) (Fongwa, Nandy, Yang, & Hays, 2015). The data sheet asked about age, marital status, education, work status, income, health insurance, source of getting treatment, weight, height, awareness of having HBP and/or diabetes, smoking history and self‐rated health. Blood pressure was directly measured for all participants using the correct arm size BP cuff. On arrival for the focus group interview, participants were allowed to rest in a chair for about five minutes prior to PI or RA taking and recording their blood pressure using an aneroid sphygmomanometer. The FATS questionnaire consists of 18 facilitators and barriers to adherence items with an internal consistency reliability of 0.78 in a sample of 70 AA women (Fongwa et al., 2015).

#### 3.2.2 Interview guide

A semi‐structured interview guide was used with the following questions: (a) From your point of view, what is the meaning of a support group? Probes for this question included the following: (i) In terms of managing your HBP, what would a support group mean? (ii) What does support group mean in terms of your life, your HBP, in facing challenges and worries in your life and caring for yourself? (b) What things would make you join a HBP support group? (c) What things might prevent you from attending a HBP support group? and (d) What important factors should be considered in forming support groups?

#### 3.2.3 Conduct of the focus group session

After participants signed the informed consent, each focus group session was started and audiotape‐recorded. The participants were seated around a table, and each was asked to choose a desired first name (to protect their identity) that was written on a visible name tag during the focus group session. Participants were informed that the focus group discussion would be summarized by the investigators and confidentiality would be protected. The focus groups were conducted in English. At the start of each session, participants were informed that it was a one‐time meeting, they would be asked to talk about the meaning of a HBP support group, facilitators and barriers to attending a support group on HBP for AA women and the factors to consider in forming a support group. The study participants were told that there were no right or wrong answers to the questions. In addition, if a participant were to remember someone else's experience who is not part of the group session, the participant could tell the group provided the person's name was not mentioned. Participants were instructed to speak clearly and one at a time to ensure we heard their valuable ideas.

#### 3.2.4 Characteristics of focus group moderator

The PI was the focus group moderator. She is an experienced health services researcher affiliated with a major university school of nursing in the western United States. The RA's role during the focus group discussion was to take notes of the proceedings, serving as a backup for the tape‐recorded interviews.

### 3.3 Data processing and analysis

Descriptive statistics were computed for the responses to the demographic and clinical data sheet and FATS questionnaire using SPSS Version 21. The study participants' body mass index (BMI) was calculated from the weight in pounds divided by height in inches squared and multiplied by a conversion factor of 703 (Centers for Disease Control & Prevention [CDC], 2011). The blood pressure readings were categorized according to the JNC‐7 (Chobanian et al., 2003) guidelines. This paper reports the results of the demographics and clinical data of the study participants and the focus group interviews.

Focus group transcripts were separately reviewed line‐by‐line and content analysed by two coders—the focus group moderator and the RA who had been trained in qualitative research. Answers to the questions were used as preliminary categories. Later, themes were created from the categories to formally represent the collected data. Concurrent coding and analysis continued until unique contents were no longer identified. The two coders compared their categories and themes, and disagreements were resolved through discussion and consensus (Ryan & Bernard, 2003).

### 3.4 Techniques to enhance trustworthiness of the study

We addressed the criteria that Lincoln and Guba (1985) enunciated to ensure trustworthiness of the study: credibility, transferability, dependability and confirmability. Credibility, the believability of the findings, was enhanced using member checks, prolonged engagement and observation and triangulation. During the group discussions, the moderator instituted member checks with focus group participants to confirm their inputs. For instance, the moderator's use of statements like “please, is this what you meant?” provided the participants to verify their opinions and perceptions. In addition, the 1.5–2 hr of duration of the focus groups allowed for prolonged engagement and observation of study participants. The assistant moderator's notes on the conduct and the proceedings of the study along with the group interactions served as a backup to the audiotape recording of the focus group sessions. Triangulation involved the use of other data sources: the study findings were compared with related studies and professional articles on support groups and social support.

Transferability refers to the degree of “fittingness” or suitability of the study findings into contexts outside the study situation (Lincoln & Guba, 1985). The diversity of our study participants' demographic background characteristics supports this criterion. Similarly, we documented the meanings of support groups to enable others to make judgements of the transferability of the study findings.

Dependability is the accounting of the process of the inquiry while confirmability refers to the accounting of the products of the inquiry. To satisfy these two criteria, we created an audit trail, documenting the study proposal, the steps taken before, during and after the focus groups—the recruitment of study participants, the conduct of the focus group interviews, the role of the assistant moderator, along with her notes of the focus group proceedings, the coding and analysis of the interview transcripts, the working drafts of the emerging conceptual framework and the final version of the derived conceptual framework. The audit trail attests to the dependability and confirmability of the study (Lincoln & Guba, 1985).

## 4 RESULTS

### 4.1 Characteristics of study participants

Twenty‐six AA women who were receiving treatment for HBP participated in the focus group interviews. Table 1 shows their socio‐demographic and clinical data. Study participants ranged in age from 42–72 years with a mean of 57 (*SD* 9). Slightly over half (57%) were single, followed by 23% married. Thirty per cent obtained some college education but slightly over 15% each finished high school, business/trade school or associate degree. Thirty‐five per cent were disabled, 19% each had part‐time work or was retired, 15% were unemployed and only 8% worked full time. Fifty per cent of the study participants earned less than $10,000, followed by nearly a fourth (23%) earning between $10,000–$19,000; 12% each earned between $20,000–29,999 and $30,000 or more. In addition, 39% had government insurance (Medicare and Medical/Medicaid) while 27% held no insurance, 15% owned commercial health insurance and 19% had missing data. The participants were evenly split in getting their health care from the research site and other agencies.

**Table 1:** Demographic and Clinical Characteristics of the 26 Study Participants (African American Women with Hypertension)

Characteristics	Number	Percentage*	Range	Mean	Standard deviation
Age in years			42–72	56.69	8.86
Marital status					
Single	15	58			
Married	6	23			
Divorced	3	12			
Widowed	2	8			
Education					
<High school graduate	4	15			
High school/GED	2	8			
Business/trade school	4	15			
Some college	8	31			
Associate degree	4	15			
Bachelor's degree	3	12			
Graduate level degree	1	4			
Work status					
Full time	2	8			
Part time, < 32 hr per week	5	19			
Unemployed	4	15			
Disabled	9	35			
Retired	5	19			
Missing	1	4			
Household income					
<$10,000	13	50			
10,000 to 19.999	6	23			
20,000 to 29.999	3	12			
30,000 or more	3	12			
Missing	1	4			
Health insurance					
Government	10	39			
Commercial	4	15			
Do not have insurance	7	27			
Missing	5	19			
Sources of getting treatment					
This research study site	13	50			
Other	13	50			
Weight in pounds			130–290	204.32	38.36
Height in inches			59–95	65.1	6.68
Underweight	2	8			
BMI (18–24.9)	1	4			
BMI: (25–29.99)	7	27			
BMI: (30 and more)	16	62			
Blood pressure: systolic: Normal: <120/< 80 mm Hg	5	19			
Pre‐hypertension: 120–139 or 80–89	8	31			
Hypertension (Stages I and II): 140–159 or 90–99 and ≥ 160 or ≥100	12	46			
Missing	1	4			
Blood pressure: diastolic					
Normal	11	42			
Pre‐hypertension	5	19			
Hypertension	9	35			
Missing	1	4			
Told by doctor has hypertension					
Yes	14	54			
No	11	42			
Missing	1	4			
Report on blood sugar					
Diabetes	9	35			
Oral medication	5	19			
Insulin dependent	4	15			
Normal	15	58			
Missing	2	8			
Smoking history					
Never smoked	7	27			
Stopped smoking	8	31			
Missing	11	42			
Entire life	18	69			
Now					
1–5 sticks per day	9	35			
6–10 sticks per day	3	12			
Half a pack per day	2	8			
Health status					
Poor	6	23			
Fair	7	27			
Good	9	37			
Very good	2	8			
Excellent	1	4			
Missing	1	4			
Health status compared with others					
Poor	4	15			
Fair	8	31			
Good	10	39			
Very good	1	4			
Excellent	2	8			
Missing	1	4			

Note

Rounded up percentages.

The participants' mean weight was 204 pounds (*SD* 38) and mean height was 65 inches (*SD* 7). BMI values indicated that about 27% were overweight (BMI, 25–29.9) while 62% of the participants were obese (BMI, 30+). Only one (4%) participant had normal BMI. The systolic blood pressure measurements showed that 19% had normal pressure. Thirty‐one per cent had pre‐hypertension pressure. Nearly half of the study participants registered systolic hypertension Stage 1 and Stage 2. On the other hand, 42% had normal diastolic pressures, followed by hypertensive pressure (35%) and pre‐hypertensive pressure of 19%. These BP readings were based on JNC‐7 blood pressure guidelines (Chobanian et al., 2003). Both JNC‐7 and the recent American College of Cardiology/American Heart Association (ACC/AHA) BP standards (Whelton et al., 2017) have normal BP values as <120/80 mm/Hg. But in the recent standards, “elevated” BP lies between systolic of 120–129 and diastolic is equal to or higher than 80 mm/Hg. Pre‐hypertension in JNC‐7 systolic BP values range is 120–139 mm/Hg. Therefore, because of the changed range for “elevated” BP in the recent ACC/AHA standards, many more of the 39% participants with JNC‐7 pre‐hypertension in this study would fall in the Stage I HBP category and the total number of those with Stages I and II HBP increased from the observed 34%**.**

Slightly more than half (53%) were told by their doctors that they had hypertension; the rest did not learn of their disease from their doctors. Slightly over half (57%) of the study participants indicated normal blood sugar and the rest (34%) reported diabetes of whom slightly above half took oral medication and slightly less than half were insulin‐dependent. Most (69%) had smoked cigarettes their entire lives, with half of them still continuing to smoke. Good, fair and poor health were reported, respectively, by 35%, 27% and 23% of the sample. Comparing their health status with others, 38% were in good health, 31% in fair health, 15% in poor health and the remainder were in very good health or in excellent health.

### 4.2 Meaning of HBP support groups

No participant had any problem answering questions about what a support group means. Participants' comments on the meaning of support groups identified four component categories or themes. The first meaning referred to the information giving/knowledge sharing of support groups, as reflected in the statement below:

A support group is where we exchange information about an issue we have with our health or other circumstance, … help us get positive results from challenges we are going to encounter, seen somebody else in the same situation… just encourage each other to overcome those difficulties… instead of suffocating yourself, you talk to somebody; stuff you don't even know about, you learn, what they do to handle theirs and how they eat.

The second meaning captured the emotional or psychological support provided by support group members. This meaning relates to the empathy shown by peers who have common experiences about the nature of HBP and the difficulty in adhering to lifestyle changes and the treatment regimen. In addition, this meaning conveys being valued, esteemed and cared for. The statements below epitomize this theme:

A support group is a place to find out … you've got someone like you to be able to understand your situation… don't feel isolated and feel like you're the only one going through…and knowing there are others like you….…sitting here…and relaxed … I was feeling uphill when I came here and she is laughing and smiling and now I feel a lot better and can breathe, So it made a change and I think … a support group is wonderful.

The third meaning was instrumental support from support groups. It denotes providing tangible help in problem‐solving. Tangible help may include doing chores such as grocery shopping and cooking a meal or driving to a doctor's appointments. It also includes helping in accessing Medicare or Medi‐Cal [Medicaid], public transport, unemployment benefits, governmental financial assistance and other community health and social services. A direct quote below and supported by group interactions illustrates this meaning:

I'm not a good cook. So, I need like a support group so somebody can say, well … this is how you make this healthier meal like this. I've always chosen work; run from the kitchen; I am not creative. I need somebody who is good to support me in cooking…

The fourth meaning indicated the coaching obtained from support groups. It signifies that support group members inspire, encourage, motivate and help each other carry out what they already know about the treatment and control of HBP—changing lifestyles and adhering to medication. This support group function is also known as health coaching. The quote below demonstrates this meaning:

… as a group, learn and teach one another about something different, exchange information … dietary ideas good for HBP… we are not supposed to eat or we should be eating. Anyone with a plan … everyone needs support to implement the tools they need to keep their BP controlled.

In sum, support groups denote a special gathering of peers that enables (a) the sharing of information and knowledge; (b) emotional and psychological support for each other; (c) the giving of tangible help in solving problems; and (d) the encouragement, coaching, motivation and carrying out of healthy behaviours and treatment adherence.

### 4.3 Factors facilitating joining support groups

The factors facilitating the women's joining support groups that emerged during the focus groups were inextricably linked with the meanings ascribed to support groups. These factors were indivisibly conjoined with the functional aspects of support groups: allowing information sharing about HBP—diet, physical activity, managing stress, HBP medication; providing emotional and psychological support such as assurance/affirmation about side effects of medications; offering tangible help with problems such as preparing meals; and coaching to carry out lifestyle changes and adherence to treatment regimen such as taking a walk to lower BP and when to eat what.

### 4.4 Factors that may prevent people from joining support groups

The participants identified several barriers to joining support groups. These barriers include work schedule that includes weekdays, emergency situations, location, scheduling conflicts like a doctor's appointment or wrong time of the day and family obligations such as child care. Other barriers participants discussed were as follows: (a) cost of bus fare or other transportation‐related costs and (b) negative attitudes of some support group members. Cited examples of negative attitudes included fighting during the meeting, people not getting along, negative comments and judgmental attitudes of some support group members. Additionally, the study participants stated that they would no longer join the group when they no longer learn something useful, making it a waste of time.

### 4.5 Important factors for consideration in forming support groups

#### 4.5.1 Timing, scheduling and frequency of group sessions

The timing, scheduling and frequency of group meetings were deduced from responses to the question, “What are the things that would make it easier to attend a support group?” The participants expressed their preferences on this factor as follows: (a) frequency of group sessions—monthly or twice a month; (b) weekdays and/or Saturdays in late morning or late afternoon and evening; (c) two sessions (early in the day time for those who do not work and evening for workers); and (d) short meeting because people are busy.

#### 4.5.2 Expectations/norms

The participants recommended that a set place and time for the support group sessions be designated ahead of time, and the support group facilitator needs to control group interactions. So few people do not dominate the group process, and the support group sessions are conveniently located with nearby free parking.

#### 4.5.3 Environmental climate

The participants described this factor in several ways: (a.) members' respect for one another (e.g. commit to attend on time by signing a pledge, listen to each other); (b) exciting group sessions make members look forward to return; (c) serving healthy snacks that model what people with HBP should eat; and (d) all members should care for one another and feel comfortable in the group regardless of people's educational attainment.

### 4.6 Conceptual model

**Figure 1:**
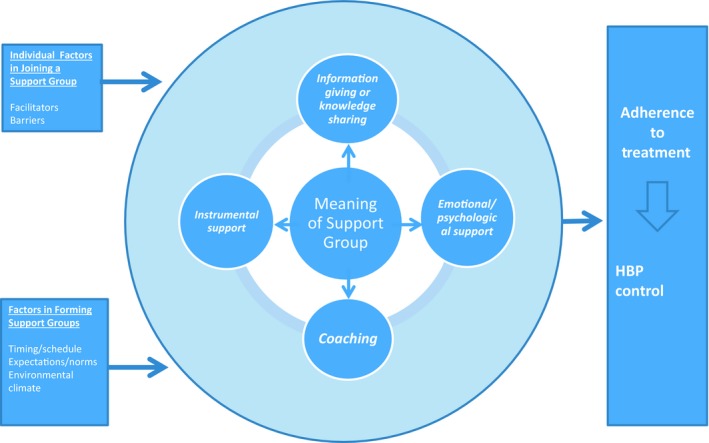
Model of high blood pressure support groups for African American women

We synthesized the results of the focus group interviews into a conceptual model (Figure 1). The centre of the model depicts the meaning of support groups which embodies the four themes: information giving/knowledge sharing, emotional or psychological support, instrumental support and coaching. The model shows the individual factors that facilitate or hinder the joining of support groups and the factors to be considered in constituting HBP support groups. Together, these factors directly influence the embodied meanings of support groups, which, in turn, influence adherence to treatment and BP control.

## 5 DISCUSSION

This study investigated the meaning of support groups and generated a conceptual model, illuminating the features of support groups that influence the adherence of AA women to HBP treatment (Figure 1). The model posits that the meaning of a support group embodies information giving/knowledge sharing, emotional or psychological support, instrumental support and coaching. In addition, the model suggests that two factors influence the execution and actualization of these support group functions: the elements that facilitate or hinder individuals joining support groups and the factors considered in forming these groups. The effective execution of the support group functions can lead to improved adherence to HBP treatment, which in turn can bring about better HBP control.

### 5.1 The meaning of support groups

The first meaning of support groups—information giving/knowledge sharing—is in line with the premise that interaction and engagement among participants in small groups can improve hypertension awareness, education and treatment in individual patients and in their communities. This functional meaning of support groups is congruent with the study findings of Shaya et al. (2013), who evaluated the effects of education administered in social networks on the improvement of HBP in 248 AAs compared with 193 historical controls who received usual care. Participants in the study formed small groups with peers and attended monthly HBP education sessions, focusing on disease education, prevention and treatment. The results showed that clustering patients in social networks around HBP education had a positive impact on managing HBP in minority populations and may help address cardiovascular health disparities. Exchange of information is facilitated in support groups because of trust and reciprocity. Smith and Christakis (2008) noted that health behaviour decisions are often made by groups. Social network interactions positively influence self‐efficacy that is needed to maintain healthy lifestyle and adherence to treatment (Smith & Christakis, 2008).

The second meaning ascribed to the emotional/psychological function of support groups in the adherence to treatment and control of HBP is supported by several studies. In a cross‐sectional design study using a randomized sample of 513 patients to determine the influence of psychosocial factors in the adherence to drug therapy in hypertensive people, Sandoval et al. (2014) found that non‐adherence was associated with high levels of emotional stress and depression. Additionally, in a Tunisian cross‐sectional study examining the links between blood pressure imbalance (fluctuations from expected normal ranges) and psychosocial factors, unbalanced blood pressure was significantly linked with poor drug adherence and depression (Masmoudi et al., 2010). Furthermore, in a Cape Town study to test the effect of an adherence support intervention delivered through text messages on blood pressure control and adherence to medication in patients with hypertension, the success of the intervention coincided with participant's readiness for change; the text messages provided practical and emotional support for improving adherence behaviours (Leon, Surrender, Bobrow, Muller, & Farmer, 2015).

The third meaning uncovered by the study involves instrumental support, also referred to as tangible support such as providing material aids or behavioural assistance (Sherborne & Hays, 1990; Sherbourne & Stewart, 1991). Given that most participants in the study had limited education and a household income of < $10,000.00, the instrumental function of support groups resonates with them. In a study on medication adherence beliefs of community‐dwelling hypertensive AAs, Lewis, Askie, Randleman, and Shelton‐Dunston (2010) found limited financial resources to be associated with behavioural control, suggesting the relevance of instrumental support among poor AAs with HBP.

The fourth meaning refers to coaching among members of support groups to maintain their health by changing lifestyles and adherence to medication to control their HBP. Also known as health coaching, it refers to helping patients get needed knowledge, skill, tools and confidence to actively take part in their own care for the purpose of achieving self‐identified health goals (Bennet et al., 2009). Health coaching has been successfully used among low‐income people as an intervention to improve providers' communication skills and stimulate patients' ability to take care of themselves with resultant positive health outcomes (Cooper et al., 2011).

In examining the study participants' perceived meaning of support groups, we noted a salient feature which suggests that each of the four meanings is highly linked with each other, especially when they emanate from the same group source (Semmer et al., 2008). This link insinuates that an emotional element—caring for the well‐being of support group members—undergirds the actualization of these support group functions, thereby generating an emotional meaning for the support of group members.

### 5.2 Facilitators and barriers to joining support groups and factors in forming groups

The study participants acknowledge that the four functional meanings of support groups serve as the facilitators for their joining them. In contrast, time conflicts, negative attitudes of support group members, transportation costs and the perceived uselessness of the group sessions constitute practical barriers to joining support groups. Hence, organizers of support groups need to consider these practical factors and the characteristics of potential members and incentives to create an environment that fosters effectively functioning support groups.

### 5.3 The conceptual model of support groups: Links with social support and resource theory of social exchange

In this study, the meanings AA women with HBP attributed to support groups correspond to similar concepts that social support theory articulates. Social support theory suggests several types of social support emanating from interpersonal relationships (Sherbourne & Stewart, 1991, p. 705): (a) emotional support which involves caring, love and empathy; (b) instrumental support (referred to by many as tangible support); (c) information, guidance or feedback that can provide a solution to a problem; (d) appraisal support which involves information relevant to self‐evaluation; and (e) social companionship, which involves spending time with others in leisure and recreational activities.

Support groups are a distinct source of social support. The study's conceptual framework on support groups in the context of AA women with HBP surfaced three out of the five types of support articulated in the theory of social support: information giving/knowledge sharing support, emotional/psychological support and instrumental support. In addition, the derived conceptual framework surfaced coaching as a function of support groups.

Inherent in the types of social support are the classes of resources exchanged in interpersonal relationships. Foa and Foa (2012, p. 16) classified these exchanged resources into six classes: (a) love, which is an expression of affectionate regard, warmth or comfort; (b) status, which indicates an evaluative judgement that conveys prestige, regard or esteem; (c) information, which includes advice, opinions, instruction or enlightenment but excludes those behaviours that could be classed as love or status; (d) money, any coin, currency or token that has some standard unit of exchange value; (e) goods are tangible products, objects or materials; and (f) service which involves activities that affect the body or belongings of a person that often constitute labour for another. The meanings of support groups uncovered in this study indicate the exchange of information (knowledge sharing), service (instrumental support, coaching) and affection (emotional/psychological support). Other studies on support groups have shown that of the six resources, love, status and information are regularly exchanged (Brown et al., 2014).

### 5.4 Strengths, limitations and future research

A strength of the study stems from the use of qualitative research employing focus groups in developing a conceptual framework of support groups in the context of AA women with HBP. This contextualized conceptualization of support groups as a source of social support grounds it in real life, in contrast to most of the deductive theoretical perspectives of social support (Hupcey, 1998; Williams, Barclay, & Schmied, 2004). As such, this study contributes to the literature on support groups as a convincing strategy to increase adherence to recommended treatment, specifically in hypertensive AA women. The findings provide a basis for creating effective support groups and testing their feasibility to increase adherence to treatment and improve HBP control. A limitation of the study is its small sample size of AA women. Because of the ethnic and gender characteristics of the small sample size, the findings may not be generalized to other populations groups. However, we recommend that future studies replicate the study with larger sample sizes of diverse patient population groups. Similarly, we suggest that future studies also investigate the potential pitfalls of support groups and how to mitigate them.

## 6 CONCLUSIONS AND IMPLICATIONS

This study generated a conceptual model derived from the uncovered meanings of support groups, the factors that facilitate or hinder joining support groups, and the factors to consider in forming them. The study suggests the need to test the feasibility of support groups as an intervention to increase adherence to treatment regimens and improve HBP control among hypertensive people, and ascertain the specific contribution of each functional meaning of support groups.

## CONFLICT OF INTEREST

The authors have no conflict of interest to declare regarding this study.
